# The function of PROTOPORPHYRINOGEN IX OXIDASE in chlorophyll biosynthesis requires oxidised plastoquinone in *Chlamydomonas reinhardtii*

**DOI:** 10.1038/s42003-019-0395-5

**Published:** 2019-05-03

**Authors:** Pawel Brzezowski, Brigitte Ksas, Michel Havaux, Bernhard Grimm, Marie Chazaux, Gilles Peltier, Xenie Johnson, Jean Alric

**Affiliations:** 1 Aix Marseille Université, CNRS, CEA, Institut de Biosciences et Biotechnologies Aix-Marseille, Laboratoire de Bioénergétique et Biotechnologie des Bactéries et Microalgues, CEA Cadarache, 13108 Saint-Paul-lez-Durance, France; 2 Humboldt-Universität zu Berlin, Institut für Biologie/Pflanzenphysiologie, 10115 Berlin, Germany; 3 Aix Marseille Université, CNRS, CEA, Institut de Biosciences et Biotechnologies Aix-Marseille, Laboratoire d’Ecophysiologie Moléculaire des Plantes, CEA Cadarache, 13108 Saint-Paul-lez-Durance, France

**Keywords:** Molecular biology, Cell biology

## Abstract

In the last common enzymatic step of tetrapyrrole biosynthesis, prior to the branching point leading to the biosynthesis of heme and chlorophyll, protoporphyrinogen IX (Protogen) is oxidised to protoporphyrin IX (Proto) by protoporphyrinogen IX oxidase (PPX). The absence of thylakoid-localised plastid terminal oxidase 2 (PTOX2) and cytochrome *b*_*6*_*f* complex in the *ptox2 petB* mutant, results in almost complete reduction of the plastoquinone pool (PQ pool) in light. Here we show that the lack of oxidised PQ impairs PPX function, leading to accumulation and subsequently uncontrolled oxidation of Protogen to non-metabolised Proto. Addition of 3(3,4-Dichlorophenyl)-1,1-dimethylurea (DCMU) prevents the over-reduction of the PQ pool in *ptox2 petB* and decreases Proto accumulation. This observation strongly indicates the need of oxidised PQ as the electron acceptor for the PPX reaction in *Chlamydomonas reinhardtii*. The PPX-PQ pool interaction is proposed to function as a feedback loop between photosynthetic electron transport and chlorophyll biosynthesis.

## Abstract (web summary)

Pawel Brzezowski et al. demonstrated existence of a link between chlorophyll biosynthesis and photosynthetic electron transport in *Chlamydomonas reinhardtii*. The lack of oxidised plastoquinone pool impairs function of PROTOPORPHYRINOGEN IX OXIDASE, leading to accumulation of non-metabolised protoporphyrin IX.

## Introduction

The tetrapyrrole biosynthesis (TBS) pathway leads to biosynthesis of chlorophyll, heme, and siroheme, which are indispensable components of cell metabolism, including energetic processes, such as chloroplast-localised photosynthesis and mitochondrial respiration. In photosynthetic organisms, several enzymatic steps of TBS lead to the biosynthesis of protoporphyrin IX (Proto), which is a common substrate for ferrochelatase (FeCh) and Mg-chelatase (MgCh), two enzymes at the TBS branching point, dedicated to biosynthesis of heme and chlorophyll, respectively. Biosynthesis of Proto is catalysed by protoporphyrinogen IX (Protogen) oxidase (PPX in *C. reinhardtii*, alias PPOX), which removes six electrons and protons from Protogen^1^. However, no information is available on which component accepts the electrons from Protogen oxidation in eukaryotic photosynthetic organisms.

In eukaryotic organisms PPOX belongs to the FAD-containing HemY-type protein family^2^ and in plants it is encoded by two nucleus-localised homologous genes, *PPOX1* and *PPOX2*. PPOX1 is targeted exclusively to plastids, providing Proto for heme and chlorophyll synthesis^3^, while PPOX2 was found in plastid envelope and mitochondria in spinach^4^. However, *N. tabacum* PPOX2 was shown to be solely a mitochondrial protein^5^. In *C. reinhardtii*, PPX is encoded by a single gene and was shown to be targeted exclusively to plastids^3^. Interactions of PPOX with other TBS enzymes, regulatory proteins, or electron acceptors, have not been reported so far.

Photosynthesis relies on a balanced linear electron transfer between photosystem II (PSII), cytochrome *b*_*6*_*f* (cyt *b*_*6*_*f*), and photosystem I (PSI), producing O_2_ at the PSII donor side and reducing NADP^+^ at the acceptor side of PSI in the light. The plastoquinone (PQ) serves as the electron carrier between PSII and cyt *b*_*6*_*f*. In darkness, the linear electron transfer is inactive, but PQ is reduced non-photochemically to plastoquinol (PQH_2_) by NAD(P)H-dehydrogenase in a process called chlororespiration^6,7^. Plastid terminal oxidase (PTOX), located on the stromal side of the thylakoid membrane, utilises its di-iron centre to oxidise PQH_2_ in conjunction with reduction of oxygen to water^8^. Thus, the PQ pool in the *ptox2* mutant is mostly reduced even in the dark^9^. The plastid-localised *PetB* gene encodes cyt *b*_*6*_, a component of the cyt *b*_*6*_*f* complex^10^. The *ptox2 petB* double mutant of *C. reinhardtii* shows a completely photochemically reduced PQ pool in light^9^, due to the electron flow from PSII and a blockage in the linear electron transfer.

Based on the study of *ptox2 petB*, we show that the deficiency in oxidised PQ leads to impairment in TBS, with a pronounced accumulation of Proto, which results from compromised function of PPX. Inhibition of an enzyme usually induces accumulation of the substrate and depletion of the product of the reaction. However, in the case of PPX, it was demonstrated previously that its substrate Protogen does not accumulate because it is non-specifically oxidised to Proto, which accumulates as an end-product^11–14^.

## Results

### DCMU treatment increases light tolerance in *ptox2 petB*

**Fig. 1:**
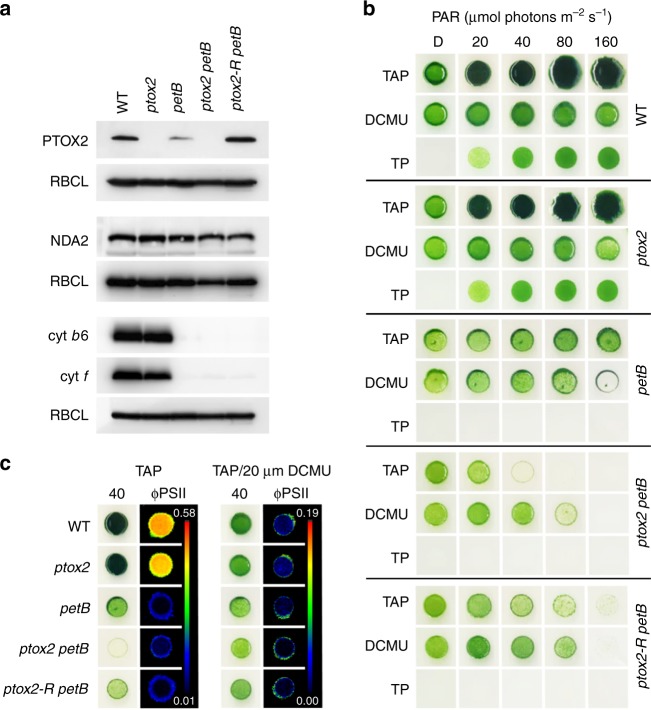
Biochemical and physiological analysis of *ptox2 petB*. **a** Western-blot analysis of PTOX2 and cyt *b*_*6*_ content in wild type (WT), *ptox2*, *petB*, *ptox2 petB*, and the *ptox2 petB* rescued with wild-type version of PTOX2 (*ptox2-R petB*). NDA2 was used as the control for the PTOX2 content, while cyt *f* was used as an indicator of the cyt *b*_*6*_*f* formation; RBCL was used as the loading control. Full images of the detected chemiluminescent signal are available in Supplementary Fig. 6. **b** Light sensitivity and photosynthetic capacity in mutants and WT control examined on TAP or TP in dark or increasing light conditions, with or without the addition of DCMU, and after 7 days exposure to experimental conditions. **c** Representative example of the chlorophyll fluorescence measurements in cells grown in 40 µmol photons m^−2^ s^−1^ on TAP without or with addition of DCMU; quantum yield of PSII (ΦPSII) parameter was used to demonstrate photochemical quenching in cells treated with DCMU (WT, *ptox2*) or to show the blockage in electron transfer due to the absence of cyt *b*_*6*_*f*

Although two genes encode PTOX in *C. reinhardtii*, PTOX2 was demonstrated to be the major oxidase involved in chlororespiration^9^. To demonstrate the photosynthetic electron transport (PET) capacity in our mutant strains, selected protein accumulation was determined in *ptox2*, *petB*, *ptox2 petB*, and the double mutant rescued with the wild-type version of PTOX2, designated *ptox2-R petB*. The *ptox2* mutant is completely devoid of PTOX2, while *petB* lacks cyt *b*_*6*_ (Fig. 1a). Consequently, *ptox2 petB* is deficient both in PTOX2 and cyt *b*_*6*_. Because cyt *b*_*6*_ is an essential subunit of the cyt *b*_*6*_*f*, the lack of *PetB* leads to the absence of cyt *b*_*6*_*f* and it was shown that the synthesis of cyt *f*, another component of cyt *b*_*6*_*f*, depends on the presence of cyt *b*_*6*_/subunit IV (*PetD*) precomplex^15^. Thus, in the present study cyt *f* was used as an additional control, to confirm the absence of cyt *b*_*6*_*f* (Fig. 1a). The type II NAD(P)H dehydrogenase (NDA2) is a component involved in chlororespiration in *C. reinhardtii*^16^. As demonstrated by immunoblot, the NDA2 content was similar in all of the mutants examined here (Fig. 1a), which indicates that the chlororespiration process is affected only due to the absence of PTOX2.

The photosynthetic phenotype of the mutants was determined on agar-solidified photoautotrophic medium (tris-phosphate, TP) and compared to growth on heterotrophic medium (tris-acetate-phosphate, TAP). The growth of *ptox2* was similar to WT in all tested conditions (Fig. 1b). However, due to the blockage of electron transfer in PET (Fig. 1c and Supplementary Fig. 1), mutants lacking cyt *b*_*6*_*f* are not able to grow on TP (Fig. 1b). The *ptox2 petB* mutant showed increased light sensitivity on TAP, compared to single *ptox2* or *petB*, or rescued *ptox2-R petB*. The light intensity of 40 µmol photons m^−2^ s^−1^ arrested growth of *ptox2 petB*, while other strains were still able to grow at two times stronger light intensities (Fig. 1b).

In TAP medium, the DCMU treatment affected growth of photosynthetic WT or *ptox2*, and increased light sensitivity of non-photosynthetic *petB* and *ptox2-R petB*. Surprisingly however, DCMU increased light tolerance of *ptox2 petB*, which grew on TAP at up to 80 µmol photons m^−2^ s^−1^, i.e., two times more than in the absence of DCMU (Fig. 1b). DCMU blocks electron transport at the acceptor side of PSII, observed as a decrease of ΦPSII (Fig. 1c), leading to charge recombination in PSII and generation of ^1^O_2_^17^. Thus, the increased light tolerance of the double *ptox2 petB* mutant does not reflect a released inhibition of PSII (see control in Fig. 1c) and, generally, it cannot be explained by the direct effect of DCMU treatment on PET.

### Accumulation of Proto in *ptox2 petB* is prevented by DCMU

**Fig. 2:**
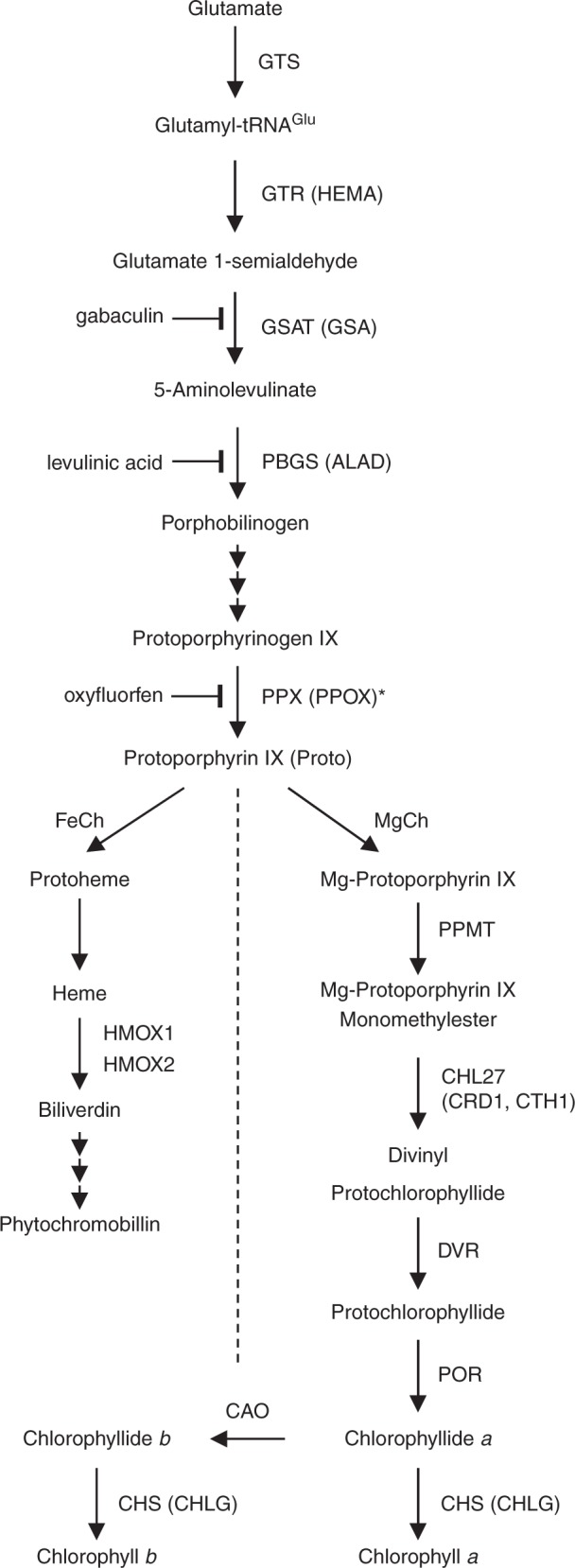
Schematic representation of the tetrapyrrole biosynthesis pathway. The protoporphyrinogen IX oxidase (PPX, alias PPOX) is marked by an asterisk. Inhibition of glutamate 1-semialdehyde aminotransferase (GSAT) by gabaculin, porphobilinogen synthase (PBGS) by levulinic acid, and PPX by oxyfluorfen is indicated. Multiple enzymatic steps leading to the conversion of porphobilinogen to protoporphyrinogen IX (Protogen), as well as subsequent steps of heme catabolism from biliverdin to formation of phytochromobilin are not shown in detail

**Fig. 3:**
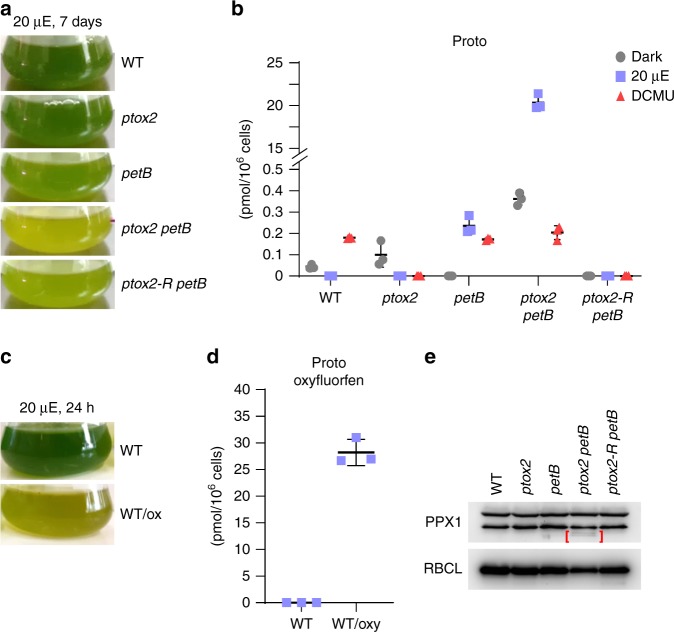
Visible pigmentation phenotype and Proto accumulation, due to the impairment in PPX function. **a** Representative samples of the cell liquid cultures of the mutants compared to wild type (WT). The *ptox2 petB* mutant demonstrated green/yellow pigmentation with additional brownish discoloration, characteristic for accumulating Proto^27^. **b** Proto accumulation in *ptox2 petB* in dark and after exposure to 20 µmol photons m^−2^ s^−1^ light. Addition of DCMU decreases Proto levels in *ptox2 petB* in the same light conditions. **c** Representative samples of WT liquid culture treated with oxyfluorfen. Note the similarity between the WT/ox and *ptox2 petB* without any chemical treatment. **d** Proto accumulation in WT treated with oxyfluorfen and shift from dark to 20 µmol photons m^−2^ s^−1^ light for 24 h. **e** PPX content analysis in mutant strains compared to WT did not show any major differences, except that two additional lower molecular weight and faint bands were detected in *ptox2 petB*, marked by brackets. RBCL was used as the loading control. Full images of the detected chemiluminescent signal are available in Supplementary Fig. 6. The HPLC analyses were performed in biological triplicates (*n* = 3); horizontal bars represent the calculated mean, vertical error bars represent the standard deviation. The source data underlying the graphs is included in the Supplementary Table 1

The TBS pathway consists of several highly-regulated steps (Fig. 2). The disturbance of any of these steps usually causes accumulation or deficiency in intermediates and affects the content of the end-products, resulting in altered pigmentation. When grown in TAP-liquid cultures (Fig. 3a), or upon prolonged growth on agar-solidified TAP (not visible on Fig. 1b), the general appearance of *ptox2 petB* was different than *ptox2*, *petB*, or wild type. The double mutant showed a pale green/yellow phenotype, with a brownish precipitate accumulating in the media (Fig. 3a), which was identified as Proto (Supplementary Fig. 2a). Treatment of *ptox2 petB* with gabaculin, which blocks one of the early steps in TBS, *i.e*. glutamate 1-semialdehyde aminotransferase (GSAT, Fig. 2), prevented accumulation of Proto in *ptox2 petB* (Supplementary Fig. 2b).

The pigment content, including the TBS intermediates and end-products (Fig. 2) were determined by High Pressure Liquid Chromatography (HPLC) in *ptox2 petB, ptox2*, *petB*, *ptox2-R petB*, and wild type. Cultures were grown in TAP either in dark or at 20 µmol m^−2^ s^−1^ light. Additional samples in the same light conditions were treated with DCMU. Proto accumulated in *ptox2 petB* >86-fold compared to *petB*, while it was not detectable in wild type or *ptox2* (Fig. 3b). Interestingly, treatment with DCMU prevented accumulation of Proto in the media (Supplementary Fig. 2b) and decreased Proto content in the *ptox2 petB* cells to values observed in *petB* or wild type (Fig. 3b).

To test whether Proto accumulation can be also observed in other mutant lines with over-reduced PQ pool, the Proto content was determined in the double mutant devoid of PTOX2 and plastocyanin, *ptox2 pcy*. Over-reduction of the PQ pool in *ptox2 pcy* in the light (Supplementary Fig. 3a) was similar to that recorded in *ptox2 petB* (Supplementary Fig. 1). Spectrometric analysis revealed lower chlorophyll content in *ptox2 pcy* (Supplementary Fig. 3b) and higher Proto to chlorophyll ratio, compared to *ptox2* (Supplementary Fig. 3c). This indicates that Proto accumulation results directly from the lack of oxidised PQ. Because non-photosynthetic mutants devoid of cyt *b*_6_*f* or plastocyanin do not synthetise ATP in the light, while certain enzymes of the TBS pathway were shown to require ATP^18–21^, or to carry putative phosphorylation sites^22–24^, we used the non-photosynthetic ATP-deficient *fud50* mutant^25,26^ as an additional control strain. Proto to chlorophyll ratio was similar in *fud50* and *ptox2* (Supplementary Fig. 3c).

The accumulation of phototoxic Proto in photosynthetic eukaryotes is considered to be due to a dysfunction of either MgCh, FeCh, or PPOX, e.g. as it was previously observed in *chli1* mutants of *C. reinhardtii*^27^, transgenic lines with diminished FeCh2 expression^28^ or PPOX-deficient^29^ mutants of *N. tabacum*, respectively. The analysis of the steady-state levels of the MgCh product, Mg-protoporphyrin (MgProto), revealed no deficiency in *ptox2 petB*. In all tested conditions, MgProto content in *ptox2 petB* was similar or even exceeded values determined for wild type (Supplementary Fig. 4a).

To investigate further if enzymes downstream from MgCh were responsible for the Proto-accumulating phenotype in *ptox2 petB*, e.g., by causing a backup of the metabolic flow through TBS, substrates and products of MgProto methyltransferase (PPMT, alias CHLM; Fig. 2), MgProto monomethylester (MgProtoME) cyclase (CRD1 or CTH1, alias CHL27, here designated as cyclase; Fig. 2), or protochlorophyllide (Pchlide) oxidoreductase (POR; Fig. 2), were determined. Particularly the cyclase would be a good candidate to be regulated by the redox reactions involving quinones, because just like PTOX^30,31^ or mitochondrial AOX^8,32–34^, it contains a typical consensus domain for a di-iron-binding site^35–37^. Indeed, it was reported that the PQ pool acts as an electron acceptor/donor for the cyclase reaction in *Arabidopsis thaliana* and *Hordeum vulgare* L.^38^. However, besides Proto, none of the quantified TBS intermediates showed an apparent deficiency or accumulation in *ptox2 petB*, compared with wild type, *ptox2*, *petB*, or *ptox2-R petB* (Supplementary Fig. 4).

These results suggested that Proto was not accumulating in response to a deregulation of the downstream steps of TBS, but directly due to an impaired function of the enzyme responsible for Proto synthesis, PPX. Several reports demonstrated Proto accumulation due to inhibition and deficiency in PPX activity^39–41^. To determine the phenotype of impaired PPX, the wild-type strain was treated with the PPX inhibitor oxyfluorfen^42,43^, which resulted in a pale-green/yellowish phenotype (Fig. 3c) and accumulation of Proto already after 24 h in TAP-liquid culture exposed to 20 µmol m^−2^ s^−1^ light (Fig. 3d). Thus, the inhibition of the PPX activity in wild type by oxyfluorfen resulted in a similar Proto accumulation as in *ptox2 petB* without chemical treatment (Fig. 3b).

To get a deeper insight into the effect of the lack of PTOX2 and cyt *b*_*6*_*f* complex, more in-depth analysis of TBS intermediates, end-products, and selected pigments were performed in the double mutant in comparison to wild type treated with oxyfluorfen. It was determined that the entire TBS pathway was deregulated in the wild-type cells treated with oxyfluorfen as well as in *ptox2 petB* (Supplementary Fig. 4). Oxyfluorfen-treated cells accumulated Zn-protoporphyrin (ZnProto), which was not found in the absence of the PPX inhibitor. ZnProto might be formed from Proto, which accepts divalent cations, due to the massive accumulation of this intermediate following oxyfluorfen treatment. It has been observed before that FeCh has a high affinity not only for Fe^2+^, but also for Co^2+^, Zn^2+^, Ni^2+^, or Cu^2+^, leading to the formation of the respective metalloporphyrins in vitro, although with FeCh-inhibitory consequences^44^. Thus, the Zn^2+^ chelation in our experiment in vivo might be due to Fe^2+^ becoming a limiting factor in protoheme biosynthesis (see Fig. 2 for the reference). Indeed, heme levels were lower in *ptox2 petB* compared to other strains grown in the light (Supplementary Fig. 4h), as well as in oxyfluorfen-treated wild type, compared to non-treated cells (Supplementary Fig. 4h). Because it was demonstrated that phytoene desaturase, an enzyme involved in carotenoid biosynthesis, depends on PQ^45,46^, the content of *β*-carotene was also determined in *ptox2 petB* and wild type treated with oxyfluorfen. Both strains showed a similar decrease in *β*-carotene levels (Supplementary Fig. 4g).

Subsequently, the PPX content was determined in *ptox2*, *petB*, *ptox2 petB*, and the rescued *ptox2-R petB*. The antibody against PPX immunoreacted with two proteins with an apparent molecular weight of 55 and 59 kDa, as it was previously reported in spinach^4^ and tobacco^5^. In plants, these protein bands were previously associated with two isoforms, which are either exclusively localised in plastids (PPOX1) or in plastids and mitochondria (PPOX2)^4,47,48^. Interestingly, although *C. reinhardtii* possesses only one *PPX1* gene, two immune-reacting protein bands were also detected, consistently with previous work of van Lis and coworkers^3^, but with independently-developed PPX antibody. All strains tested contained similar levels of these two immune-reacting PPX variants, indicating that one protein band potentially corresponds to posttranslationally modified form of PPX, with possible degradation products detected as two additional faint bands, below 55 kDa in *ptox2 petB* (Fig. 3e).

Changes in steady-state levels of tetrapyrrole metabolites can be caused by deregulated or impaired 5-aminolevulinic acid (ALA; Fig. 2) synthesis, the rate limiting step of TBS. ALA synthesis capacity in the dark was similar in *ptox2 petB* compared to *ptox2*, *petB*, *ptox2-R petB*, and wild type(Supplementary Fig. 5a). Compared to wild type, higher ALA formation was detected in all of the mutants exposed to 20 µmol photons m^−2^ s^−1^ (Supplementary Fig. 5a), which in *ptox2* resulted in higher chlorophyll and heme content compared to wild type (Supplementary Fig. 4e, h). Exposure to 40 µmol photons m^−2^ s^−1^ decreased ALA synthesis in *petB*, *ptox2 petB*, and *ptox2-R petB* (Supplementary Fig. 5b). Thus, altered ALA synthesis rates in the mutants devoid of cyt *b*_*6*_*f* cannot be responsible for Proto accumulation in *ptox2 petB*.

Notably, DCMU treatment increased chlorophyll content in *ptox2 petB* when compared to non-treated *ptox2 petB*, which is indicative that DCMU rescues the phenotype in the double mutant, not only by diminishing Proto levels (Fig. 3b), but also by restoring the chlorophyll content (compare Supplementary Fig. 4e, f).

## Discussion

Houille-Vernes and co-workers demonstrated that *ptox2 petB* shows almost complete reduction of the PQ pool in light^9^. As shown in the present study, over-reduction of the PQ pool is accompanied by accumulation of Proto, resulting from impaired function of PPX responsible for controlled Protogen oxidation. However, because Proto is a substrate of MgCh and FeCh, we examined whether impairment in these enzymatic steps could be responsible for the phenotype in *ptox2 petB*. Hypothetically, the impairment of MgCh could be twofold. First, the over-reduced PQ pool may directly affect MgCh function. Second, in phosphoproteomics studies it was proposed that certain TBS proteins, including MgCh subunits and PPX, may be regulated by phosphorylation^22–24,49^. Protein phosphorylation was confirmed experimentally for the integral MgCh subunit CHLD of *C. reinhardtii* and *Oryza sativa*^21^, as well as for the regulatory protein GUN4 of *A. thaliana*, which is phosphorylated in the dark to halt chlorophyll synthesis^50^. In the light, neither the cyt *b*_6_*f*-deficient mutants *petB*, *ptox2 petB* nor the ATPase-deficient *fud50* mutant produce ATP in the chloroplast, but Proto accumulation was only observed in the double mutant *ptox2 petB* (compare Fig. 3b and Supplementary Fig. 3c). We therefore conclude that, although PPX activity may be regulated by phosphorylation^24,49^, it requires oxidised PQ as an electron acceptor for Protogen oxidation. We also demonstrated that MgCh function is not responsible for the Proto accumulation in *ptox2 petB*, because it did not show deficiency in MgProto (Supplementary Fig. 4a). Moreover, addition of DCMU does not increase ATP levels but prevents Proto accumulation in *ptox2 petB*. In terms of the possible FeCh impairment causing Proto accumulation in *ptox2 petB*, there is no indication that this enzymatic step requires ATP, and the heme levels are similar with or without DCMU treatment. Finally, Mg^2+^ or Fe^2+^ chelation are not redox reactions involving transfer of electrons, and it is unlikely that MgCh or FeCh activity would rely on the PQ pool status, or that they would be directly affected by DCMU. On the other hand, other components of PET upstream from PQ, particularly NDA2 involved in NAD(P)H-dependent PQ reduction, potentially might also affect the PPX activity. However, the NDA2 levels were similar in all of the strains (Fig. 1a), which demonstrated that NDA2 is not responsible for the Proto-accumulating phenotype in *ptox2 petB*.

Proto is the sole TBS intermediate accumulating in *ptox2 petB*, while measurements of the ALA synthesis capacity (Supplementary Fig. 5) showed similar trends in all of the mutants lacking cyt *b*_*6*_*f*. If the ALA synthesis capacity would have shown an increase in the double mutant, as opposed to single *ptox2* or *petB*, it might have been indicative of an elevated flow through TBS pathway in *ptox2 petB*, potentially resulting in accumulation of Proto, due to an impairment or bottleneck in steps downstream from PPX (See Fig. 2 for reference). Because ALA synthesis capacity does not trigger increased metabolic flow through TBS in *petB*, *ptox2 petB*, and *ptox2-R-petB* (Supplementary Fig. 5), Proto accumulation can be solely assigned to an impaired PPX activity. There is a certain variation in ALA synthesis capacity in 20 µmol photons m^−2^ s^−1^ light in all of the analyzed mutants (Supplementary Fig. 5a), but a clear pattern emerges from experiment performed in 40 µmol photons m^−2^ s^−1^ (Supplementary Fig. 5b). In this light condition, ALA synthesis capacity decreases in all tested non-photosynthetic mutants. Decrease in ALA might be indicative of the oxidative stress caused not only by accumulating Proto in *ptox2 petB* (Fig. 3b), but also due to the ROS resulting from the blockage in electron transfer in all of the mutants devoid of cyt *b*_*6*_*f*.

Thus, the double mutant was used to demonstrate dependence of the PPX reaction on the redox state of the PQ pool. It was shown that application of DCMU correlates with reduced Proto level in *ptox2 petB* and increased tolerance to light (Fig. 1b). It has to be noted, that there is no case in the literature describing a direct effect of DCMU on any enzymatic step of TBS. Moreover, the mode of action of this herbicide has only an indirect character in the study described here. Thus, it can also be concluded that increased light tolerance in DCMU-treated *ptox2 petB* results from diminished Proto accumulation. Furthermore, DCMU application not only decreased accumulation of Proto in *ptox2 petB* (Fig. 3b), but also rescues the chlorophyll level in this strain, bringing it back to the contents observed in *petB* or *ptox2-R petB* (compare Supplementary Fig. 4e, f). This is indicative of the restored function of PPX and biosynthesis of Proto in a more controlled fashion, which makes it accessible for MgCh.

**Fig. 4:**
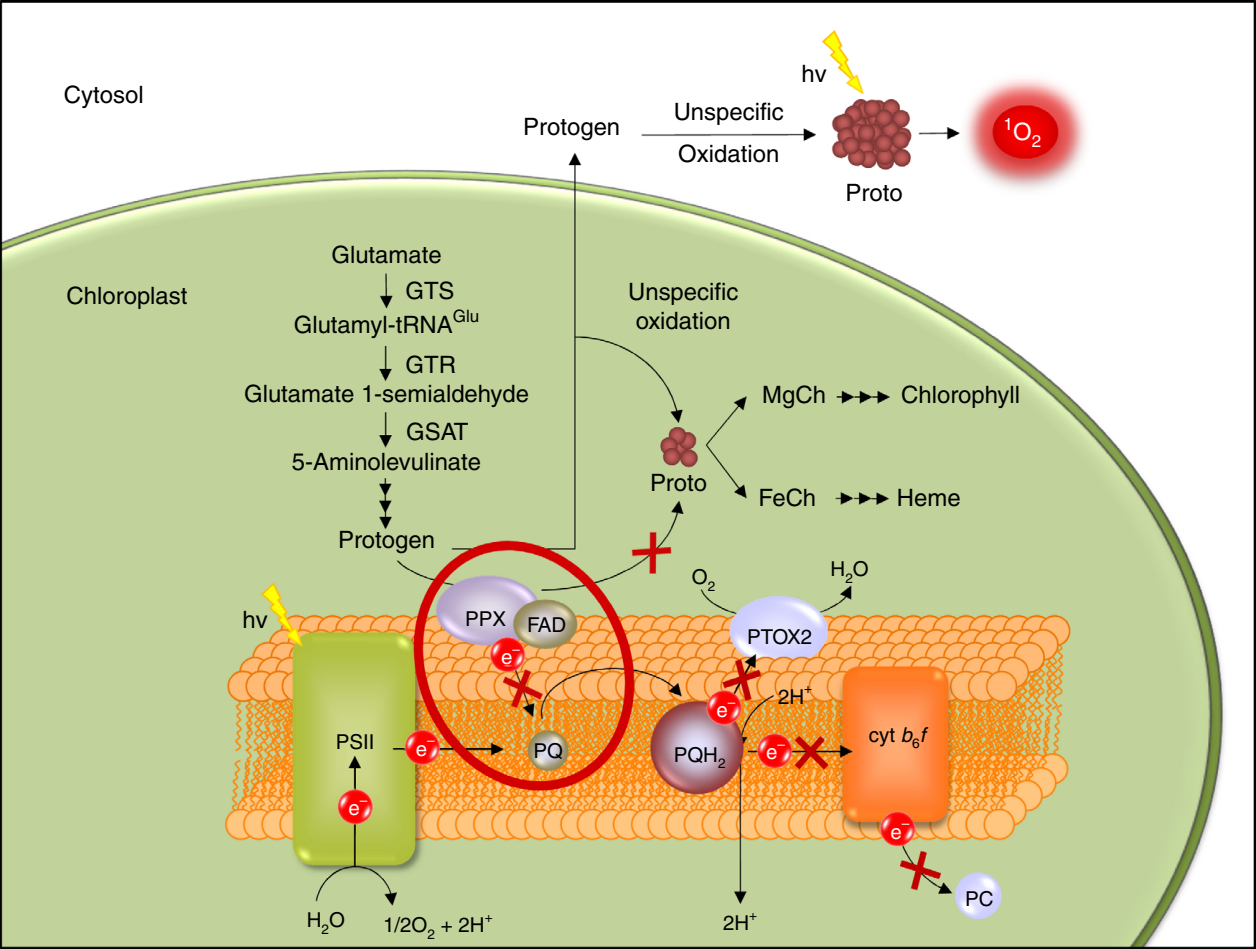
Schematic representation of a proposed model of the TBS pathway interaction with PET. In normally functioning PET, electrons derived from water are subsequently transferred from photosystem II (PSII) to plastoquinone (PQ) generating its reduced form, plastoquinol (PQH_2_). PQH_2_ is then oxidised by transferring the electrons to cyt *b*_*6*_*f* and by PTOX2. In the *ptox2 petB* mutant, the PQ pool is almost completely reduced^9^, as depicted by the greater PQH_2_ oval (PQ«PQH_2_). In such conditions, PPX cannot transfer the electrons derived from Protogen to PQ. FAD cofactor present in PPX is proposed to mediate in electron transfer from PPX to PQ. Thus, lack of the oxidised plastoquinone (PQ) leads to a dysfunction or a complete blockage of the PPX function. This in turn prevents synthesis of Proto in a controlled fashion. Subsequently, Protogen is unspecifically oxidised in the chloroplast and after sequestration in the cytosol, leading to the formation of Proto, which is inaccessible for MgCh and FeCh. Ultimately, accumulating Proto leaks out of the chloroplast and outside of the cell, which is observed as the brownish precipitate in the media. However, a small portion of the Proto formed in the chloroplast may be still available for the enzymes subsequently leading to biosynthesis of chlorophyll and heme

Following Möbius and co-workers^51^, we propose that, similarly to its bacterial counterpart HemG, algal PPX is a thylakoid membrane-bound^3^ protoporphyrinogen IX/plastoquinone oxido-reductase (Fig. 4). The six electrons and protons extracted from Protogen would be transferred to PQ to form 3 PQH_2_ molecules. In the dark, O_2_ would serve as a terminal electron acceptor via the activity of plastid terminal oxidase PTOX2 and to a lesser extent PTOX1.

By comparison of the oxyfluorfen-treated wild type with non-treated and illuminated *ptox2 petB*, we conclude that a similar mechanism is responsible for the accumulation of Proto in the double mutant. Moreover, Proto accumulation in *ptox2 petB* and oxyfluorfen-treated wild type is not the only common denominator. ZnProto might be accumulating in both strains due to the accumulation of Proto *per se*. Furthermore, alterations in the content of other intermediates showed similar patterns when *ptox2 petB* and wild type treated with oxyfluorfen were compared to their respective controls (Supplementary Fig. 4).

A complex regulatory network is responsible for transcriptional, translational, and post-translational regulation of TBS. These processes assure balanced metabolic flow through TBS pathway and an adequate supply of TBS end-products at different developmental stages and in response to changing environmental conditions^52,53^. Metabolic control ensures avoidance of accumulating tetrapyrrole intermediates, which are capable of generating reactive oxygen species (ROS) and organic radicals^54,55^. Not surprisingly, the activity of the TBS enzymes also includes redox regulation involving the ferredoxin-thioredoxin (FDX-TRX) system^56,57^ and NTRC^58,59^. While TRX-FDX derive electrons from PET in the light^60,61^, NTRC constitutes a NAD(P)H-dependent reductase^62,63^. Both systems are crucial for TBS regulation^64,65^. Additionally, a component of the cyclase, YCF54^66^ (LCAA^67^), was shown to act as the scaffolding factor for CHL27^67^ and was recently demonstrated to interact with ferredoxin-NADPH reductase (FNR1), downstream of PSI in *A. thaliana*^68^. Lack of YCF54 results in accumulation of MgProtoME and decrease of Pchlide, Chlide, and chlorophyll^68^. Thus, it was suggested that FNR1 acts as an electron donor, required for the MgProtoME cyclisation reaction^68^. Here in our work with *ptox2 petB*, we disclose a different type of regulation, upstream of PSI and cyt *b*_6_*f*, at the level of the PQ pool, more similar to the dynamic PQ pool model proposed for the cyclase regulation in *A. thaliana* and *H. vulgare*^38^. PPX-PQ pool interaction in *C. reinhardtii* is further supported by the presence of a FAD-binding domain in PPX^69^, commonly found in enzymes interacting with plastoquinone, e.g., most of the eukaryotic type II NAD(P)H dehydrogenases^70,71^ or phytoene desaturase^45,72^. It is very likely that FAD in PPX plays the role of a prosthetic group, mediating transfer of the electrons removed from Protogen to PQ, and this process is responsible for maintaining functional PPX (Fig. 4).

With increasing precision, biologists are using systems biology to correlate different, well-studied physiological processes in the cell. Not surprisingly, such correlations exist between chlorophyll biosynthesis and chlorophyll (and heme)-dependent photosynthetic processes, specifically, components of PET. The results presented here provide further evidence for an interaction between the TBS pathway and PET. This regulation of PPX activity simply relies on the availability of oxidised PQ and provides a regulatory control point at the cross-roads between chlorophyll biosynthesis and PET. Thus, in this model, the redox state of the PQ pool acts as a sensor of the electron flow in PET, determining chlorophyll requirements and adjusting its biosynthesis by modulation of PPX activity.

## Methods

### *Chlamydomonas* cultures and genetic manipulations

The wild type (Jex4), generation of *ptox2*, *petB*, and *ptox2 petB* were described elsewhere^9^. The *ptox2 pcy* double mutant was generated by crossing *ptox2* (*mt*^*-*^)^9^ with *pcy* (*mt*^*+*^)^73^, while *fud50* was described in Woessner et al. (1984) and Lemaire et al. (1988). For the rescue of the *petB* phenotype in *ptox2 petB*, the *PTOX2* cDNA was provided by F.A. Wollman (IBPC, Paris). The *PTOX2* transcript was amplified by PCR using primers 5′-CATATGATGCTCGCGGCCAGGCAGC-3′, and 5′-GAATTCTCAGCGGCGGGGCGC-3′, carrying *Nde*I and *EcoR*I restriction sites, respectively. Primers were designed to include exclusively the *PTOX2* coding sequence. Obtained fragment was ligated into *Nde*I/*EcoR*I site of the pGenD2 vector carrying 5′-sequence and 3′-sequence of *PSAD*^74^, obtained from Chlamydomonas Centre (University of Minnesota). The resulting vector was named PTOX2/pGenD2. The cell transformation was conducted by electroporation. Following the introduction of PTOX2/pGenD2 into *ptox2 petB*, the successful transformation was confirmed by chlorophyll fluorescence measurements (Supplementary Fig. 1) and immunoblot with PTOX2 antibody (Fig. 1a), the rescued strain was named *ptox2-R petB*. Because *PSAD* carries a strong promoter, transformation of *ptox2 petB* with PTOX2/pGenD2 in fact resulted in higher PTOX2 content in *ptox2-R petB* compared to wild type (Fig. 1a).

All strains were cultivated either heterotrophically in dark or mixotrophically in light on TAP or photoautotrophically in Tris-phosphate (TP, devoid of acetate). Most of the experiments were performed in dark or in 20 µmol photons m^−2^ s^−1^, unless otherwise indicated.

### Protein analysis

The standard methods were applied for the protein extraction, except that the western-blot analyses were performed on the membrane enriched fraction. The cell pellet was resuspended in TBS buffer consisting of 500 mM Tris and 150 mM NaCl, pH 7.5 and sonicated. Following centrifugation, protein were extracted in 400 µL buffer containing 56 mM Na_2_CO_3_, 56 mM DTT, 2% SDS, 12% sucrose and 2 mM EDTA, and separated on 12% SDS-PAGE, followed by transfer to nitrocellulose membrane. PTOX2 was detected using purified antipeptide-raised antibody against *C. reinhardtii* PTOX2^9^. NDA2 was detected by a polyclonal rabbit antibody described in Jans et al.^16^. RBCL was detected with commercially available antibody (Agrisera). The components of the cyt *b*_*6*_*f*, *b*_*6*_ and *f* were detected with antibody raised against *C. reinhardtii* proteins. The PPX content was determined following the immunoreaction with antibody raised against recombinant PPOX1 of *N. tabacum*^5^. The chemiluminescence signal was detected using G:Box Chemi XL system (Syngene) after application of Immobilon Western HRP Substrate (Merck). Quantification of the signal was performed using GeneTools software (Syngene).

### ALA synthesis capacity

The ALA synthesis capacity was determined following 24 h inhibition of porphobilinogen synthase (PBGS, alias ALA dehydratase, ALAD, see Fig. 2 for reference) with levulinic acid in dark, or exposure to 20 µmol photons m^−2^ s^−1^, or 40 µmol photons m^−2^ s^−1^. Extracted ALA was converted to a pyrrole using ethyl acetoacetate, and after the formation of the chromophore with aminobenzaldehyde in modified Ehrlich’s reagent, ALA derivatives were quantified spectrophotometrically at 553 nm^75,76^.

### Chlorophyll fluorescence measurements

All chlorophyll fluorescence measurements were performed using system described in Johnson^77^ allowing time-resolved chlorophyll fluorescence in response to light directly on petri dishes, equipped with 12-bits high frame rate (150 frames per second) CCD camera, which allows 100 µs sampling. The detection system is synchronised with actinic light and saturating pulses. After experimental treatment, chlorophyll fluorescence images were recorded in dark-adapted samples over the course of 6 min, with 5 saturating light pulses, to determine quantum yield of PSII (ΦPSII) and obtain chlorophyll fluorescence kinetics.

### Chemical treatment

The DCMU was dissolved in EtOH to a stock solution of 20 mM concentration and the final concentration used for the experiments was 20 µM. Thus, the final concentration of EtOH in the samples did not exceed 0.1%. The stock solution of 125 µM oxyfluorfen in DMSO was added to the wild-type culture to a final concentration of 25 nM, and cells were transferred to light. The concentration of DMSO in culture was 0.02%.

### Analysis of the TBS intermediates and end-products

The brownish filamentous precipitate accumulating in the media of illuminated *ptox2 petB* cultures was filtered through a stainless steel mesh, washed free from chlorophyll and carotenoids with methanol, followed by solubilisation of the precipitate in DMSO. Fluorescence spectrum was analysed at *λ*_em_ 550–750.

The steady-state levels of the TBS pathway intermediates and end-products were analysed by HPLC in the pellet of 1.2 × 10^8^ cells, following extraction in cold acetone/0.1 M NH_4_OH(9/1, v/v) in a three-step cycle of resuspension and centrifugation. Proto analyses were performed using Nova-Pak C18 column (Waters, 3.9 × 150 mm, 4 μm, at 20 °C), MgProto and MgProtoME using Poroshell column (Agilent, 3.0 × 150 mm, 2.7 μm, at 4 °C), with solvent A (80% H_2_O, 10% methanol, and 10% ammonium acetate (1 M, pH 7.0) and solvent B (70% acetonitrile, 20% acetone, and 40% ammonium acetate (20 mM, pH 5.16), at a flow rate 1 mL min^−1^. Fluorescence detection was conducted at *λ*_ex_ 405 nm and *λ*_em_ 637 nm for Proto, and *λ*_ex_ 420 nm and *λ*_em_ 600 nm for MgProto and MgProtoME.

Chlorophylls were separated on a Prontosil 200-3-C30 (bischoff-chromatography) column (3 μm; 250 × 4.6 mm; 21 °C) at a flow rate of 1 mL min^−1^ and eluted with a gradient of solvent A (90% acetonitrile; 10% water; 0.1% triethylamine) and solvent B (100% ethyl acetate). Detection was conducted by DAD (Agilent 1100) at an absorption wavelength of 440 nm (peak width 10 Hz; slit width 4 nm).

Heme extraction was continued from a pellet after the acetone/0.1 M NH_4_OH (9/1, v/v) extraction, in acetone/HCl/DMSO (10/0.5/2, v/v/v) in the same three-step protocol. Heme was separated on a Poroshell 120 EC-C18 (Agilent) column (2.7 μm; 100 × 3.0 mm; 30 °C) at a flow rate of 0.8 mL min^−1^ and eluted with a gradient of solvent A (water pH 3.2) and solvent B (methanol). Detection was performed by DAD (Agilent 1290) at an absorption wavelength of 398 nm (peak width 2.5 Hz; slit width 4 nm). Results obtained from the HPLC analyses of all tetrapyrroles and pigments analysed were always calculated on a per cell basis.

### Reporting summary

Further information on experimental design is available in the Nature Research Reporting Summary linked to this article.

## Supplementary information

Supplementary Information

Reporting Summary

## Data Availability

The data that supports the findings of this study are available from the corresponding author upon reasonable request.

## References

[1] Ferreira GC, Dailey HA (1988). Mouse protoporphyrinogen oxidase. Kinetic parameters and demonstration of inhibition by bilirubin. Biochem. J..

[2] Dailey TA, Dailey HA (1996). Human protoporphyrinoagen oxidase: Expression, purification, and characterization of the cloned enzyme. Protein Sci..

[3] van Lis R, Atteia A, Nogaj LA, Beale SI (2005). Subcellular localization and light-regulated expression of protoporphyrinogen IX oxidase and ferrochelatase in *Chlamydomonas reinhardtii*. Plant Physiol..

[4] Watanabe N (2001). Dual targeting of spinach protoporphyrinogen oxidase II to mitochondria and chloroplasts by alternative use of two in-frame initiation codons. J. Biol. Chem..

[5] Lermontova I, Kruse E, Mock HP, Grimm B (1997). Cloning and characterization of a plastidal and a mitochondrial isoform of tobacco protoporphyrinogen IX oxidase. Proc. Natl Acad. Sci. USA.

[6] Bennoun P (1982). Evidence for a respiratory chain in the chloroplast. Proc. Natl Acad. Sci. USA.

[7] Desplats C (2009). Characterization of Nda2, a plastoquinone-reducing type II NAD(P)H dehydrogenase in chlamydomonas chloroplasts. J. Biol. Chem..

[8] Berthold DA, Stenmark P (2003). Membrane-bound diiron carboxylate proteins. Annu. Rev. Plant. Biol..

[9] Houille-Vernes L, Rappaport F, Wollman FA, Alric J, Johnson X (2011). Plastid terminal oxidase 2 (PTOX2) is the major oxidase involved in chlororespiration in Chlamydomonas. Proc. Natl Acad. Sci. USA.

[10] Buschlen S, Choquet Y, Kuras R, Wollman FA (1991). Nucleotide sequences of the continuous and separated petA, petB and petD chloroplast genes in Chlamydomonas reinhardtii. FEBS Lett..

[11] Jacobs JM, Jacobs NJ (1993). Porphyrin accumulation and export by isolated barley (Hordeum-Vulgare) plastids—effect of diphenyl ether herbicides. Plant Physiol..

[12] Lee HJ, Duke MV, Duke SO (1993). Cellular-localization of protoporphyrinogen-oxidizing activities of etiolated barley (Hordeum-Vulgare L) leaves—relationship to mechanism of action of protoporphyrinogen oxidase-inhibiting herbicides. Plant Physiol..

[13] Matsumoto H, Kashimoto Y, Warabi E (1999). Basis for common chickweed (Stellaria media) tolerance to oxyfluorfen. Pestic. Biochem. Physiol..

[14] Duke SO, Becerril JM, Sherman TD, Matsumoto H (1991). Photosensitizing porphyrins as herbicides. Acs Symp. Ser..

[15] Kuras R, Wollman FA (1994). The assembly of cytochrome b6/f complexes: an approach using genetic transformation of the green alga Chlamydomonas reinhardtii. EMBO J..

[16] Jans F (2008). A type II NAD(P)H dehydrogenase mediates light-independent plastoquinone reduction in the chloroplast of Chlamydomonas. Proc Natl Acad Sci USA.

[17] Krieger-Liszkay A, Rutherford AW (1998). Influence of herbicide binding on the redox potential of the quinone acceptor in photosystem—II. Relevance to photodamage and phytotoxicity. Biochemistry.

[18] Jensen PE, Gibson LCD, Hunter CN (1999). ATPase activity associated with the magnesium-protoporphyrin IX chelatase enzyme of *Synechocystis* PCC6803: evidence for ATP hydrolysis during Mg2+ insertion, and the MgATP-dependent interaction of the ChlI and ChlD subunits. Biochem. J..

[19] Jensen PE, Reid JD, Hunter CN (2000). Modification of cysteine residues in the ChlI and ChlH subunits of magnesium chelatase results in enzyme inactivation. Biochem. J..

[20] Ikegami A (2007). The CHLI1 subunit of Arabidopsis thaliana magnesium chelatase is a target protein of the chloroplast thioredoxin. J. Biol. Chem..

[21] Sawicki A, Zhou S, Kwiatkowski K, Luo M, Willows RD (2017). 1-N-histidine phosphorylation of ChlD by the AAA(+) ChlI2 stimulates magnesium chelatase activity in chlorophyll synthesis. Biochem. J..

[22] Lohrig K, Muller B, Davydova J, Leister D, Wolters DA (2009). Phosphorylation site mapping of soluble proteins: bioinformatical filtering reveals potential plastidic phosphoproteins in *Arabidopsis thaliana*. Planta.

[23] Reiland S (2009). Large-scale Arabidopsis phosphoproteome profiling reveals novel chloroplast kinase substrates and phosphorylation networks. Plant Physiol..

[24] Sugiyama N (2008). Large-scale phosphorylation mapping reveals the extent of tyrosine phosphorylation in Arabidopsis. Mol. Syst. Biol..

[25] Woessner JP (1984). Molecular and genetic analysis of the chloroplast ATPase of chlamydomonas. Plant Mol. Biol..

[26] Lemaire C, Wollman FA, Bennoun P (1988). Restoration of phototrophic growth in a mutant of Chlamydomonas reinhardtii in which the chloroplast atpB gene of the ATP synthase has a deletion: an example of mitochondria-dependent photosynthesis. Proc Natl Acad Sci USA.

[27] Brzezowski P (2016). Mg chelatase in chlorophyll synthesis and retrograde signaling in Chlamydomonas reinhardtii: CHLI2 cannot substitute for CHLI1. J. Exp. Bot..

[28] Papenbrock J (2001). Impaired expression of the plastidic ferrochelatase by antisense RNA synthesis leads to a necrotic phenotype of transformed tobacco plants. Plant J..

[29] Lermontova I, Grimm B (2006). Reduced activity of plastid protoporphyrinogen oxidase causes attenuated photodynamic damage during high-light compared to low-light exposure. Plant J..

[30] Fu A, Park S, Rodermel S (2005). Sequences required for the activity of PTOX (IMMUTANS), a plastid terminal oxidase: in vitro and in planta mutagenesis of iron-binding sites and a conserved sequence that corresponds to Exon 8. J. Biol. Chem..

[31] Berthold DA, Andersson ME, Nordlund P (2000). New insight into the structure and function of the alternative oxidase. Biochim. Biophys. Acta.

[32] Siedow JN, Umbach AL, Moore AL (1995). The active site of the cyanide-resistant oxidase from plant mitochondria contains a binuclear iron center. FEBS Lett..

[33] Moore AL, Umbach AL, Siedow JN (1995). Structure-function relationships of the alternative oxidase of plant mitochondria: a model of the active site. J. Bioenerg. Biomembr..

[34] Berthold DA, Voevodskaya N, Stenmark P, Graslund A, Nordlund P (2002). EPR studies of the mitochondrial alternative oxidase. Evidence for a diiron carboxylate center. J. Biol. Chem..

[35] Pinta V, Picaud M, Reiss-Husson F, Astier C (2002). *Rubrivivax gelatinosus* acsF (previously orf358) codes for a conserved, putative binuclear-iron-cluster-containing protein involved in aerobic oxidative cyclization of Mg-protoporphyrin IX monomethylester. J. Bacteriol..

[36] Walker CJ, Castelfranco PA, Whyte BJ (1991). Synthesis of divinyl protochlorophyllide. Enzymological properties of the Mg-protoporphyrin IX monomethyl ester oxidative cyclase system. Biochem. J..

[37] Moseley J, Quinn J, Eriksson M, Merchant S (2000). The Crd1 gene encodes a putative di-iron enzyme required for photosystem I accumulation in copper deficiency and hypoxia in *Chlamydomonas reinhardtii*. EMBO J..

[38] Steccanella V, Hansson M, Jensen PE (2015). Linking chlorophyll biosynthesis to a dynamic plastoquinone pool. Plant Physiol. Biochem..

[39] Matringe M, Camadro JM, Labbe P, Scalla R (1989). Protoporphyrinogen oxidase as a molecular target for diphenyl ether herbicides. Biochem. J..

[40] Yamato S, Ida T, Katagiri M, Ohkawa H (1995). A tobacco soluble protoporphyrinogen-oxidizing enzyme similar to plant peroxidases in their amino acid sequences and immunochemical reactivity. Biosci. Biotechnol. Biochem..

[41] Becerril JM, Duke SO (1989). Protoporphyrin IX content correlates with activity of photobleaching herbicides. Plant Physiol..

[42] Sandmann, G. & Böger, P. Accumulation of protoporphyrin IX in the presence of peroxidizing herbicides. *Z. Naturforsch*. **43c**, 699–704 (1988).

[43] Lee JJ, Matsumoto H, Ishizuka K (1992). Light involvement in oxyfluorfen-induced protoporphyrin IX accumulation in several species of intact plants. Pestic. Biochem. Physiol..

[44] Hunter GA, Sampson MP, Ferreira GC (2008). Metal ion substrate inhibition of ferrochelatase. J. Biol. Chem..

[45] Norris SR, Barrette TR, DellaPenna D (1995). Genetic dissection of carotenoid synthesis in Arabidopsis defines plastoquinone as an essential component of phytoene desaturation. Plant Cell.

[46] Carol P (1999). Mutations in the Arabidopsis gene IMMUTANS cause a variegated phenotype by inactivating a chloroplast terminal oxidase associated with phytoene desaturation. Plant Cell.

[47] Che FS (2000). Molecular characterization and subcellular localization of protoporphyrinogen oxidase in spinach chloroplasts. Plant Physiol..

[48] Narita S (1996). Molecular cloning and characterization of a cDNA that encodes protoporphyrinogen oxidase of Arabidopsis thaliana. Gene.

[49] Manohara MS, Tripathy BC (2000). Regulation of protoporphyrin IX biosynthesis by intraplastidic compartmentalization and adenosine triphosphate. Planta.

[50] Richter AS (2016). Phosphorylation of GENOMES UNCOUPLED 4 alters stimulation of Mg chelatase activity in angiosperms. Plant Physiol..

[51] Mobius K (2010). Heme biosynthesis is coupled to electron transport chains for energy generation. Proc. Natl Acad. Sci. USA.

[52] Mochizuki N (2010). The cell biology of tetrapyrroles: a life and death struggle. Trends. Plant. Sci..

[53] Schlicke Hagen, Richter Andreas, Rothbart Maxi, Brzezowski Pawel, Hedtke Boris, Grimm Bernhard (2015). Function of Tetrapyrroles, Regulation of Tetrapyrrole Metabolism and Methods for Analyses of Tetrapyrroles. Procedia Chemistry.

[54] Halliwell B (2006). Reactive species and antioxidants. Redox biology is a fundamental theme of aerobic life. Plant Physiol..

[55] Apel K, Hirt H (2004). Reactive oxygen species: metabolism, oxidative stress, and signal transduction. Annu. Rev. Plant. Biol..

[56] Balmer Y (2003). Proteomics gives insight into the regulatory function of chloroplast thioredoxins. Proc. Natl Acad. Sci. USA.

[57] Marchand C, Le Marechal P, Meyer Y, Decottignies P (2006). Comparative proteomic approaches for the isolation of proteins interacting with thioredoxin. Proteomics.

[58] Richter AS (2013). Posttranslational influence of NADPH-dependent thioredoxin reductase C on enzymes in tetrapyrrole synthesis. Plant Physiol..

[59] Stenbaek A, Jensen PE (2010). Redox regulation of chlorophyll biosynthesis. Phytochemistry.

[60] Lemaire SD, Michelet L, Zaffagnini M, Massot V, Issakidis-Bourguet E (2007). Thioredoxins in chloroplasts. Curr. Genet..

[61] Hanke G, Mulo P (2013). Plant type ferredoxins and ferredoxin-dependent metabolism. Plant Cell Environ..

[62] Serrato AJ, Perez-Ruiz JM, Spinola MC, Cejudo FJ (2004). A novel NADPH thioredoxin reductase, localized in the chloroplast, which deficiency causes hypersensitivity to abiotic stress in *Arabidopsis thaliana*. J. Biol. Chem..

[63] Perez-Ruiz JM (2006). Rice NTRC is a high-efficiency redox system for chloroplast protection against oxidative damage. Plant Cell.

[64] Lepistö A (2009). Chloroplast NADPH-thioredoxin reductase interacts with photoperiodic development in Arabidopsis. Plant Physiol..

[65] Richter, A. S. & Grimm, B. Thiol-based redox control of enzymes involved in the tetrapyrrole biosynthesis pathway in plants. *Front. Plant Sci*. **4**, 371 (2013). 10.3389/fpls.2013.00371 PMC3778395 24065975

[66] Hollingshead S (2012). Conserved chloroplast open-reading frame ycf54 is required for activity of the magnesium protoporphyrin monomethylester oxidative cyclase in *Synechocystis* PCC 6803. J. Biol. Chem..

[67] Albus CA (2012). LCAA, a novel factor required for magnesium protoporphyrin monomethylester cyclase accumulation and feedback control of aminolevulinic acid biosynthesis in tobacco. Plant Physiol..

[68] Herbst J, Girke A, Hajirezaei MR, Hanke G, Grimm B (2018). Potential roles of YCF54 and ferredoxin-NADPH reductase for magnesium protoporphyrin monomethylester cyclase. Plant J..

[69] Dailey TA, Dailey HA (1998). Identification of an FAD superfamily containing protoporphyrinogen oxidases, monoamine oxidases, and phytoene desaturase. Expression and characterization of phytoene desaturase of Myxococcus xanthus. J. Biol. Chem..

[70] Rasmusson AG, Geisler DA, Moller IM (2008). The multiplicity of dehydrogenases in the electron transport chain of plant mitochondria. Mitochondrion.

[71] Melo AM, Bandeiras TM, Teixeira M (2004). New insights into type II NAD(P)H:quinone oxidoreductases. Microbiol. Mol. Biol. Rev..

[72] Mayer MP, Beyer P, Kleinig H (1990). Quinone compounds are able to replace molecular oxygen as terminal electron acceptor in phytoene desaturation in chromoplasts of Narcissus pseudonarcissus L. Eur. J. Biochem..

[73] Johnson, X., Kuras, R., Wollman, F. A. & Vallon, O. Gene Hunting by Complementation of Pooled *Chlamydomonas* Mutants, in *Photosynthesis. Energy from the Sun*. (eds J. F. Allen, E. Gantt, J. H. Golbeck & B. Osmond) 1093–1099 (Springer, New York, 2007).

[74] Fischer N, Rochaix JD (2001). The flanking regions of PsaD drive efficient gene expression in the nucleus of the green alga Chlamydomonas reinhardtii. Mol. Genet. Genomics..

[75] Mauzerall D, Granick S (1956). The occurrence and determination of delta-amino-levulinic acid and porphobilinogen in urine. J. Biol. Chem..

[76] Weinstein JD, Beale SI (1985). Enzymatic conversion of glutamate to delta-aminolevulinate in soluble extracts of the unicellular green alga, Chlorella vulgaris. Arch. Biochem. Biophys..

[77] Johnson X (2009). A new setup for in vivo fluorescence imaging of photosynthetic activity. Photosynth. Res..

