# Supplementary material for: The function of PROTOPORPHYRINOGEN IX OXIDASE in chlorophyll biosynthesis requires oxidised plastoquinone in Chlamydomonas reinhardtii
Source: Commun Biol. 2019 May 3;2:159. doi: 10.1038/s42003-019-0395-5 (PMC6499784; doi:10.1038/s42003-019-0395-5)
Supplement: Supplementary file 1 — Supplementary Information [file 42003_2019_395_MOESM1_ESM.pdf]

Supplementary Information

Supplementary Figures

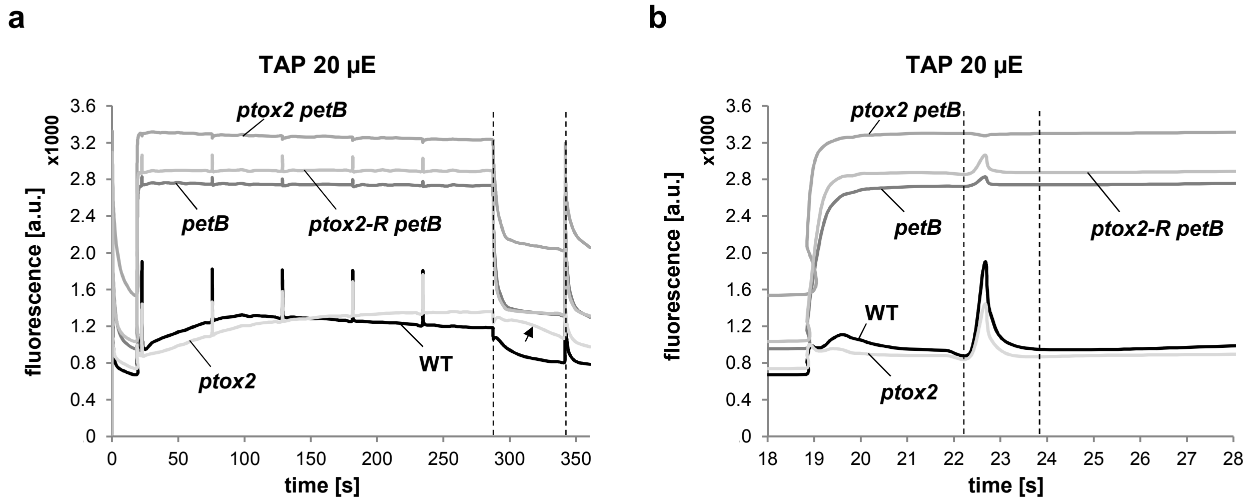

Supplementary Figure 1. Chlorophyll fluorescence kinetics. **a,** Kinetics measured over the course of 6 min. Dotted lines mark borders of the section representing the PQ relaxation (oxidation) in dark after the period of illumination, delayed relaxation in *ptox2* is indicated by arrow; *petB*, *ptox2 petB*, and *ptox2-R petB* demonstrate block in electron transfer, as indicated by high and steady chlorophyll fluorescence in actinic light. **b,** The same kinetics results but presented with higher time resolution, from 18 to 28 s. Dotted lines mark section indicating peak of the chlorophyll fluorescence rise due to the PQH_2 oxidation in light. The fluorescence peak in *ptox2* is smaller compared to wild type and might be indicative of the PTOX1 activity[1] or photochemical quenching. Mutants *petB* and *ptox2-R petB* show PTOX2 activity, confirming the successful rescue of the *petB* phenotype in the latter one. Blocked *ptox2 petB* demonstrated lack of the PQH_2 oxidation by PTOX2.

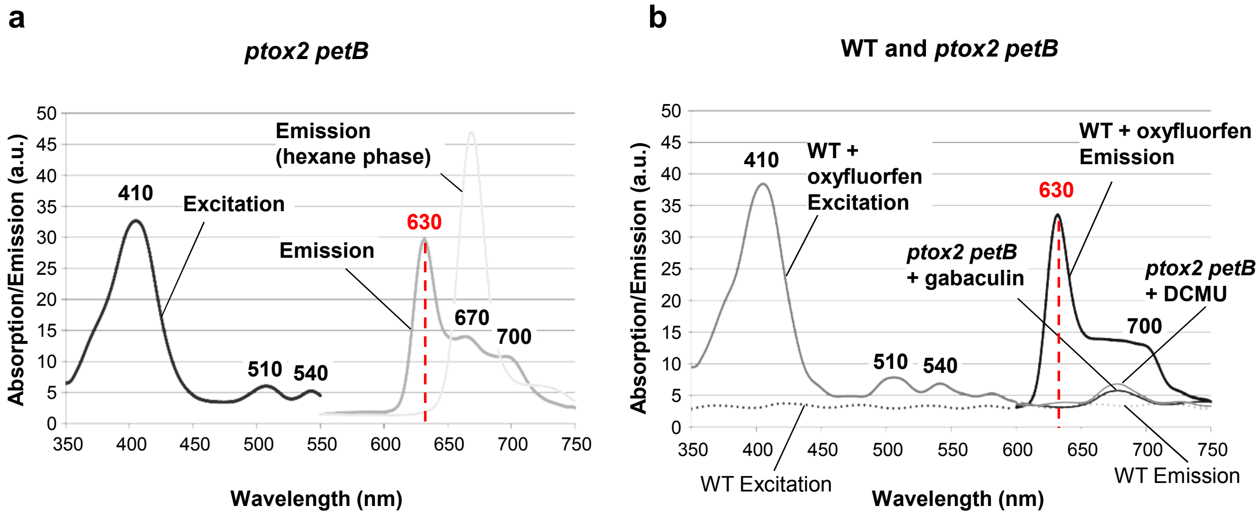

Supplementary Figure 2. Absorption and fluorescence emission spectra analysis on the DMSO-solubilized brownish precipitate in *ptox2 petB*. **a,** Excitation at λ_{ex} 410 of the *ptox2 petB* extract induced fluorescence peak at λ_{em} 630, indicating accumulation of Proto. **b,** Comparative analysis of *ptox2 petB* and WT after treatment with gabaculin and DCMU. Application of oxyfluorfen in WT shows the same excitation and emission spectrum as non-treated *ptox2 petB*, with peaks at λ_{ex} 410 and λ_{ex} 630, respectively. Treatment of *ptox2 petB* with gabaculin or DCMU abolished accumulation of Proto.

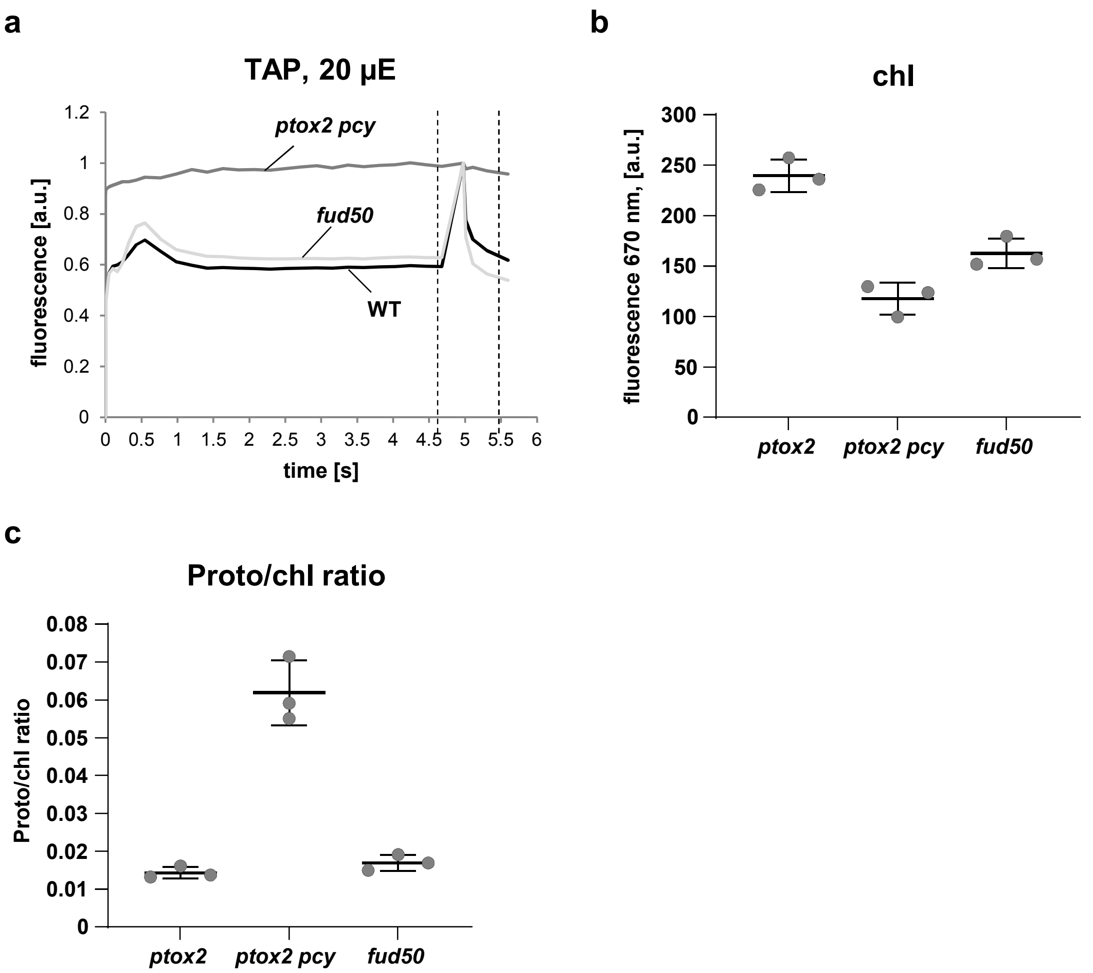

Supplementary Figure 3. Over-reduced PQ pool and Proto in *ptox2 pcy* and *fud50*. **a,** Chlorophyll fluorescence kinetics, *ptox2 pcy* demonstrated block in electron transfer, as indicated by high and steady chlorophyll fluorescence in actinic light. Dotted lines mark section indicating peak of the chlorophyll fluorescence rise due to the PQH_2 oxidation by PTOX2 in WT and *fud50*, absent in *ptox2 pcy*. **b,** chlorophyll content in *ptox2 pcy* and *fud50* compared to *ptox2*. **c,** Proto to chlorophyll ratio in analyzed mutants. Spectrometrical analysis of Proto and chlorophyll were performed in biological triplicates (*n*=3); horizontal bars represent the calculated mean, vertical error bars indicate the standard deviation.

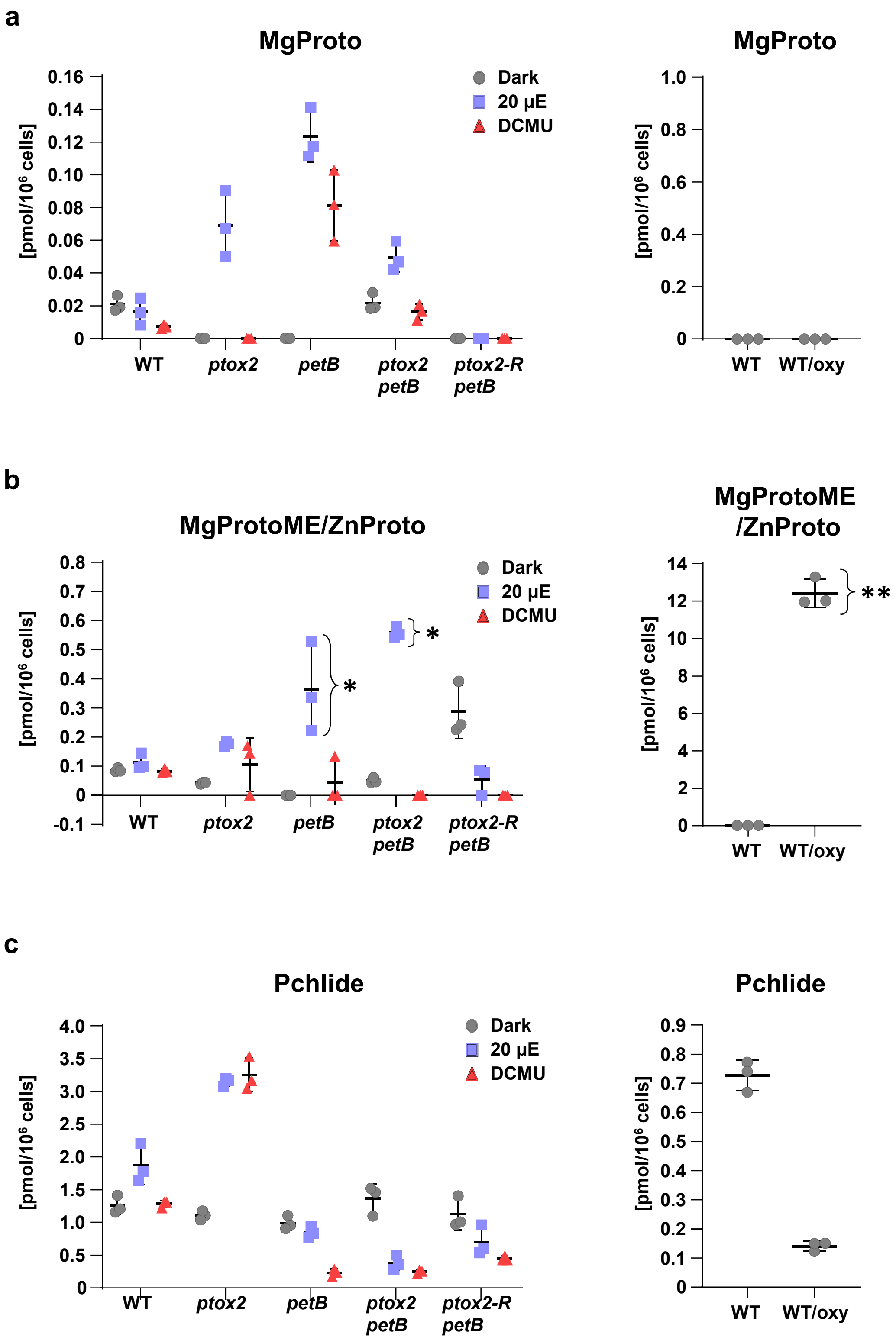
a
MgProto
[pmol/10⁶ cells]
0.16
0.14
0.12
0.10
0.08
0.06
0.04
0.02
0
Dark
20 µE
DCMU
WT
ptox2
petB
ptox2 petB
ptox2-R petB
MgProto
1.0
0.8
0.6
0.4
0.2
0
WT
WT/oxy
b
MgProtoME/ZnProto
0.8
0.7
0.6
0.5
0.4
0.3
0.2
0.1
0
-0.1
*
*
MgProtoME /ZnProto
14
12
10
8
6
4
2
0
**
c
Pchlide
4.0
3.5
3.0
2.5
2.0
1.5
1.0
0.5
0
Pchlide
0.9
0.8
0.7
0.6
0.5
0.4
0.3
0.2
0.1
0

d

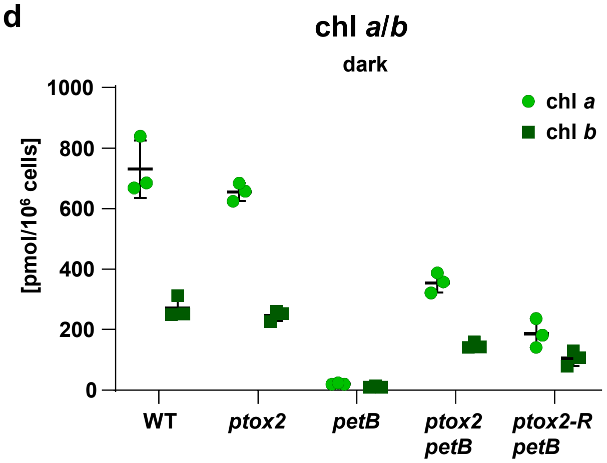
chl a/b
dark
chl a
chl b
[pmol/10⁶ cells]
0
200
400
600
800
1000
WT
ptox2
petB
ptox2 petB
ptox2-R petB

e

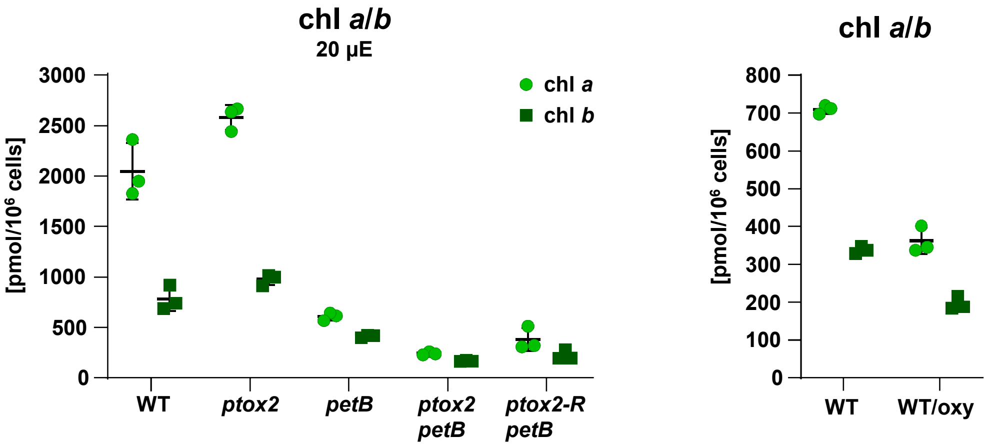
chl a/b
20 µE
chl a
chl b
[pmol/10⁶ cells]
0
500
1000
1500
2000
2500
3000
WT
ptox2
petB
ptox2 petB
ptox2-R petB
chl a/b
[pmol/10⁶ cells]
0
100
200
300
400
500
600
700
800
WT
WT/oxy

f

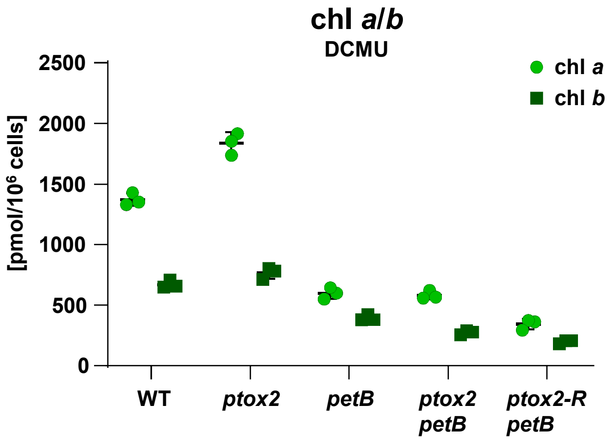
chl a/b
DCMU
chl a
chl b
[pmol/10⁶ cells]
0
500
1000
1500
2000
2500
WT
ptox2
petB
ptox2 petB
ptox2-R petB

g

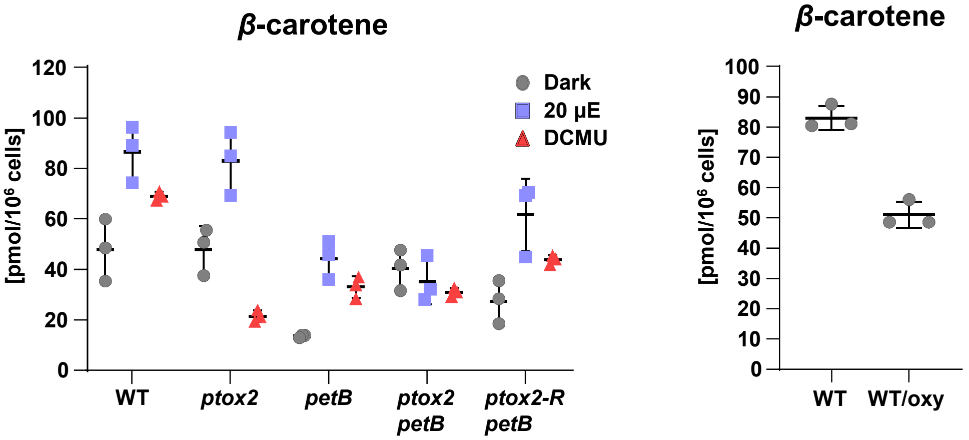
β-carotene
120
100
80
60
40
20
0
[pmol/10⁶ cells]
Dark
20 µE
DCMU
WT
ptox2
petB
ptox2
petB
ptox2-R
petB
β-carotene
100
90
80
70
60
50
40
30
20
10
0
[pmol/10⁶ cells]
WT
WT/oxy

h

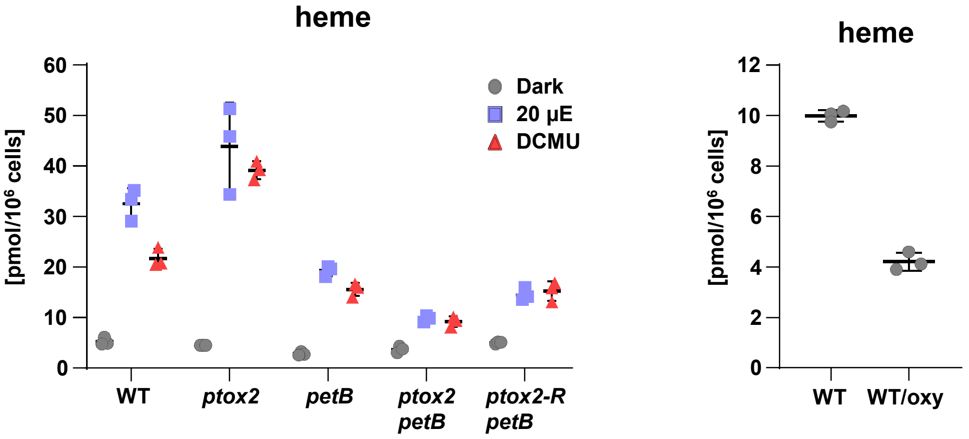
heme
60
50
40
30
20
10
0
[pmol/10⁶ cells]
Dark
20 µE
DCMU
WT
ptox2
petB
ptox2
petB
ptox2-R
petB
heme
12
10
8
6
4
2
0
[pmol/10⁶ cells]
WT
WT/oxy

Supplementary Figure 4. HPLC analysis of TBS intermediates, chlorophyll, heme, and pigments in *ptox2*, *petB*, *ptox2 petB*, rescued *ptox2-R petB*, and wild type. The experiment was performed in dark, 20 µmol photons m^{-2} s^{-1}, or in the same light conditions after addition of DCMU. Obtained results were also compared to measurements obtained for the wild type treated with 25 nM oxyfluorfen and shifted from dark to the same light condition, denoted as WT/oxy on the graphs. The experiment determining the effect of oxyfluorfen treatment was performed separately from mutants analysis. **a,** The *ptox2 petB* mutant did not show deficiency in MgProto, which otherwise might have been indicative of an impaired function of MgCh. MgProto in WT/oxy was not detectable. **b**, *petB* and *ptox2 petB* accumulated mixture of MgProtoME and ZnProto, which was confirmed by HPLC analysis with the ZnProto standard. However, the exact quantification was impossible because in our HPLC method MgProtoME and ZnProto have the same retention time and only 5 nm different fluorescence peaks. The same was observed in WT/oxy. **c,** Pchlide level was lower in double mutant compared to wild type in the same conditions, the same was observed for the WT/oxy. **d,** chlorophyll a and b were approximately 50% of the wild type level in dark. **e,** In light, chlorophyll *a* in *ptox2 petB* was nearly 12% of the wild type level, with a clear shift for chl *a*/*b* ratio, while chlorophyll in WT/oxy was 50% when compared to the respective WT. **f,** Interestingly, after DCMU treatment chlorophyll in *ptox2 petB* was similar to *petB* and *ptox2-R petB* levels, and 2 times higher compared to non-treated *ptox2 petB*. This is indicative that the DCMU treatment not only decreased Proto, but consequently increased the chlorophyll content in the double mutant. **g,** β-carotene levels showed the same trend in *ptox2 petB* and WT/oxy, when compared to corresponding wild type controls. **h,** Heme levels in dark were similar in all analyzed strains, while in light it was lower in non-photosynthetic mutants compared to *ptox2* or wild type. No major effect of DCMU on heme content was observed, but oxyfluorfen reduced heme in wild type. All experiments were performed in biological triplicates (*n*=3); horizontal bars represent the calculated mean, vertical error bars indicate the standard deviation.

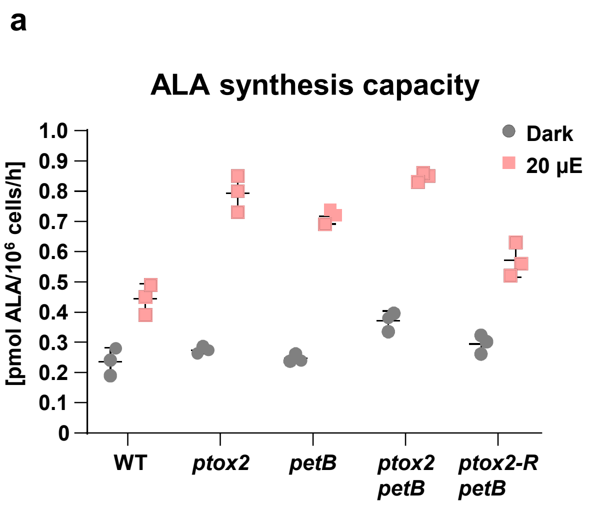

Supplementary Figure 5. Determination of 5-aminolevulinic acid (ALA) synthesis capacity in *ptox2*, *petB*, *ptox2 petB*, and *ptox2-R petB*, compared to WT. **a**, In dark and 20 µmol photons m^{-2} s^{-1} light. **b,** ALA in 40 µmol photons m^{-2} s^{-1}. All experiments were performed in biological triplicates (*n*=3); horizontal bars represent the calculated mean, vertical error bars represent the standard deviation.

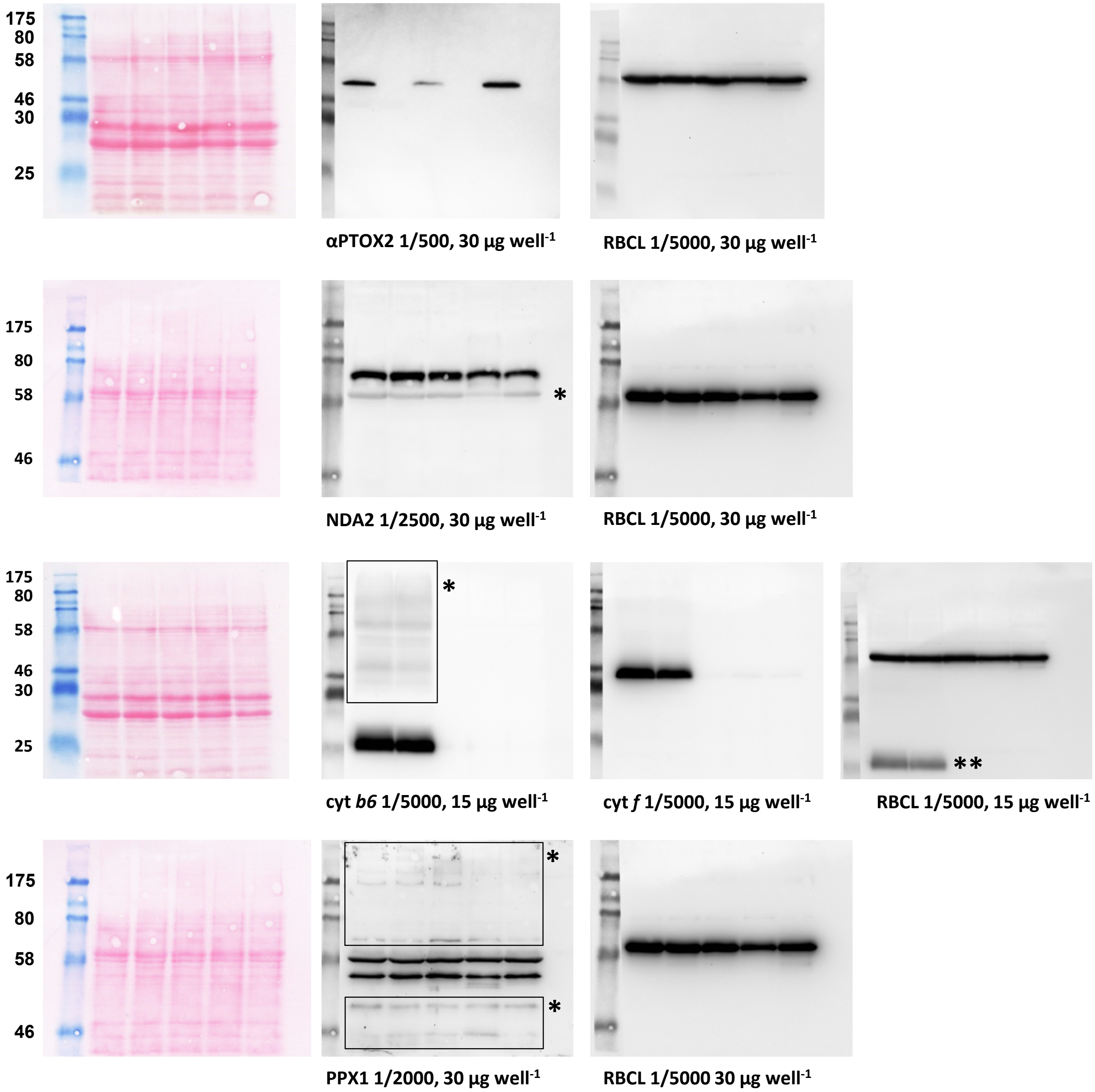

Supplementary Figure 6. Supplementary material for western blots in Figure 2a and Figure 3e. Proteins of a membrane-enriched fraction were extracted as described in Methods, followed by separation on 12% SDS-PAGE and transfer to the nitrocellulose membrane. Unspecific immunoreactions are indicated by an asterisk. Incomplete stripping of cyt *b6* antibody is indicated by a double asterisk. In all cases Prestained Protein Marker P7708 (New England Biolabs, www.neb.com) was used as a reference.

Supplementary Tables

Supplementary Table 1. Supplementary material for Figure 3b and 3d.

	Strain/triplicates	Injection volume [µL]	Area	Calculated value pmol 10^6 cells $^{-1}$
**dark**	WT-A	20	467	0.039
	WT-B	20	660	0.055
	WT-C	20	445	0.037
	ptox2-A	20	2000	0.166
	ptox2-B	20	670	0.056
	ptox2-C	20	924	0.077
	petB-A	20	0	0
	petB-B	20	0	0
	petB-C	20	0	0
	ptox2 petB-A	20	4365	0.362
	ptox2 petB-B	20	4000	0.332
	ptox2 petB-C	20	4700	0.390
	ptox2-R petB-A	20	0	0.000
	ptox2-R petB-B	20	0	0.000
	ptox2-R petB-C	20	0	0.000
**20 µE**	WT-A	20	0	0
	WT-B	20	0	0
	WT-C	20	0	0
	ptox2-A	20	0	0
	ptox2-B	20	0	0
	ptox2-C	20	0	0
	petB-A	20	2600	0.216
	petB-B	20	2520	0.209
	petB-C	20	3420	0.284
	ptox2 petB-A	5	59875	19.871
	ptox2 petB-B	5	64445	21.388
	ptox2 petB-C	5	59550	19.763
	ptox2-R petB-A	20	0	0
	ptox2-R petB-B	20	0	0
	ptox2-R petB-C	20	0	0

Continues on the next page

	Strain/triplicates	Inj. volume [µL]	Area	Calculated value pmol 10^6 cells $^{-1}$
20 µE/20 µM DCMU	WT-A	20	2150	0.178
	WT-B	20	2120	0.176
	WT-C	20	2210	0.183
	ptox2-A	20	0	0
	ptox2-B	20	0	0
	ptox2-C	20	0	0
	petB-A	20	2100	0.174
	petB-B	20	2130	0.177
	petB-C	20	2000	0.166
	ptox2 petB-A	20	2750	0.228
	ptox2 petB-B	20	2000	0.166
	ptox2 petB-C	20	2600	0.216
	ptox2-R petB-A	20	0	0
	ptox2-R petB-B	20	0	0
	ptox2-R petB-C	20	0	0
20 µE/25 nM oxyfluorfen /24 h	WT-A	20	0	0
	WT-B	20	0	0
	WT-C	20	0	0
	WT/oxy-A	2	32135	26.662
	WT/oxy-B	2	32476	26.945
	WT/oxy-C	2	37375	31.009

Raw data from the HPLC analysis and calculated values. Protoporphyrin IX (Frontier Scientific, Logan, UT, US) standard area was determined to be 7533 pmol^{-1}; total number of cells used for the extraction was normalised to 1.2 x 10^8; total extraction volume of the solution was 1.5 mL; the final value = area / 7533 / injection volume x 1500 / 120. Mean and standard deviation for each triplicate were directly calculated and graphed in statistical analysis and graphing software, GraphPad Prism 8 (GraphPad Software, San Diego, California, US).

Supplementary References

1. Houille-Vernes, L., Rappaport, F., Wollman, F.-A., Alric, J. & Johnson, X. Plastid terminal oxidase 2 (PTOX2) is the major oxidase involved in chlororespiration in Chlamydomonas. *Proceedings of the National Academy of Sciences of the United States of America* **108**, 20820-20825 (2011).
