# Supplementary material for: The function of PROTOPORPHYRINOGEN IX OXIDASE in chlorophyll biosynthesis requires oxidised plastoquinone in Chlamydomonas reinhardtii
Source: Commun Biol. 2019 May 3;2:159. doi: 10.1038/s42003-019-0395-5 (PMC6499784; doi:10.1038/s42003-019-0395-5)
Supplement: Supplementary file 2 — Reporting Summary [file 42003_2019_395_MOESM2_ESM.pdf]

nature research

Corresponding author(s): Pawel Brzezowski

Reporting Summary

Nature Research wishes to improve the reproducibility of the work that we publish. This form provides structure for consistency and transparency in reporting. For further information on Nature Research policies, see Authors & Referees and the Editorial Policy Checklist.

Statistical parameters

When statistical analyses are reported, confirm that the following items are present in the relevant location (e.g. figure legend, table legend, main text, or Methods section).

n/a	Confirmed	
☐	☒	The exact sample size (n) for each experimental group/condition, given as a discrete number and unit of measurement
☐	☒	An indication of whether measurements were taken from distinct samples or whether the same sample was measured repeatedly
☐	☒	The statistical test(s) used AND whether they are one- or two-sided<br>*Only common tests should be described solely by name; describe more complex techniques in the Methods section.*
☒	☐	A description of all covariates tested
☒	☐	A description of any assumptions or corrections, such as tests of normality and adjustment for multiple comparisons
☐	☒	A full description of the statistics including central tendency (e.g. means) or other basic estimates (e.g. regression coefficient) AND variation (e.g. standard deviation) or associated estimates of uncertainty (e.g. confidence intervals)
☒	☐	For null hypothesis testing, the test statistic (e.g. F, t, r) with confidence intervals, effect sizes, degrees of freedom and P value noted<br>*Give P values as exact values whenever suitable.*
☒	☐	For Bayesian analysis, information on the choice of priors and Markov chain Monte Carlo settings
☒	☐	For hierarchical and complex designs, identification of the appropriate level for tests and full reporting of outcomes
☒	☐	Estimates of effect sizes (e.g. Cohen's d, Pearson's r), indicating how they were calculated
☐	☒	Clearly defined error bars<br>*State explicitly what error bars represent (e.g. SD, SE, CI)*

Our web collection on statistics for biologists may be useful.

Software and code

Policy information about availability of computer code

Data collection	DNA and RNA sequence data were collected from JGI (DOE Joint Genome Institute,Walnut Creek, CA, USA, https://phytozome.jgi.doe.gov/pz/portal.html); chemiluminescence signal was detected using G:Box Chemi XL system (Syngene); chlorophyll fluorescence was determined using paradigm camera SpeedZen software, BeamBio (Johnson et al. 2009), HPLC data were collected using ChemStation software (Agilent)
Data analysis	DNA and RNA sequence data were analyzed using Vector NTI (Invitrogen); chemiluminescence signal was analyzed using G:Box Chemi XL system (Syngene); chlorophyll fluorescence was analyzed using paradigm camera SpeedZen software, BeamBio (Johnson et al. 2009), HPLC data was analyzed using ChemStation software (Agilent) and Microsoft Excel 2010. For the analysis of the HPLC and ALA synthesis capacity, the GraphPad Prism 8 (GraphPad Software, San Diego, California, US) was used to calculate statistical parameters and to produce the graphs.

For manuscripts utilizing custom algorithms or software that are central to the research but not yet described in published literature, software must be made available to editors/reviewers upon request. We strongly encourage code deposition in a community repository (e.g. GitHub). See the Nature Research guidelines for submitting code & software for further information.

Data

Policy information about availability of data

All manuscripts must include a data availability statement. This statement should provide the following information, where applicable:

- Accession codes, unique identifiers, or web links for publicly available datasets
- A list of figures that have associated raw data
- A description of any restrictions on data availability

The authors declare no restrictions on data availability and most of the data supporting the findings of this study are included in the manuscript or its supplementary material. The raw data can be made available upon reasonable request from the corresponding author. The DNA/RNA sequence is publicly available at https://phytozome.jgi.doe.gov/pz/portal.html

Field-specific reporting

Please select the best fit for your research. If you are not sure, read the appropriate sections before making your selection.

☒ Life sciences ☐ Behavioural & social sciences ☐ Ecological, evolutionary & environmental sciences

For a reference copy of the document with all sections, see nature.com/authors/policies/ReportingSummary-flat.pdf

Life sciences study design

All studies must disclose on these points even when the disclosure is negative.

Sample size	the cell concentration was determined using Multisizer 3 Particle Counter (Beckman Coulter) or hemocytometer, followed by normalization where aplicable, as indicated in the manuscript
Data exclusions	no data were excluded from the analysis
Replication	Where applicable, biological triplicates were analyzed to confirm data reproducibility. That is, the growth was conducted and experimental conditions were applied on separate cultures, cells were normalized and analyzed using basic statistical methods.
Randomization	not applicable, the cell number of a given strain (indicated in the manuscript) assures randomization
Blinding	not applicable, the cell number of a given strain (indicated in the manuscript) assures blinding

Reporting for specific materials, systems and methods

Materials & experimental systems

n/a	Involved in the study
☒	☐ Unique biological materials
☐	☒ Antibodies
☐	☒ Eukaryotic cell lines
☒	☐ Palaeontology
☒	☐ Animals and other organisms
☒	☐ Human research participants

Methods

n/a	Involved in the study
☒	☐ ChIP-seq
☒	☐ Flow cytometry
☒	☐ MRI-based neuroimaging

Antibodies

Antibodies used	PTOX2 was detected using purified antipeptide raised against C. reinhardtii PTOX2 (Houille-Vernes et al., 2011). NDA2 was detected by a polyclonal rabbit antibody described in Jans et al. (2008). RBCL was detected with commercially available antibody (AS03 037, Agrisera). The components of the cyt b6f, b6 (AS18 4169) and f (AS06 119) were detected with antibody raised against C. reinhardtii proteins (Agrisera). The PPX content was determined following the immunoreaction with antibody raised against recombinant PPOX1 of N. tabacum (Lermontova et al., 1997).
Validation	all of the antibodies used in this study were described and validated in the respective publications, or are commercially available, as indicated in the Methods section

Eukaryotic cell lines

Policy information about cell lines

Cell line source(s)	The wild type, ptox2, petB, and ptox2 petB were described in Houille-Vernes et al. (2011).
Authentication	Strains were authenticated in Houille-Vernes et al. (2011).
Mycoplasma contamination	Cell lines were not tested for mycoplasma contamination
Commonly misidentified lines (See ICLAC register)	not applicable
